# Needle tracking and segmentation in breast ultrasound imaging based on spatio-temporal memory network

**DOI:** 10.3389/fonc.2024.1519536

**Published:** 2025-01-17

**Authors:** Qiyun Zhang, Jiawei Chen, Jinhong Wang, Haolin Wang, Yi He, Bin Li, Zhemin Zhuang, Huancheng Zeng

**Affiliations:** ^1^ College of Engineering, Shantou University, Shantou, Guangdong, China; ^2^ Department of Ultrasound, Shantou Chaonan Minsheng Hospital, Shantou, Guangdong, China; ^3^ Shantou University Medical College, Shantou, Guangdong, China; ^4^ Department of Ultrasound, Shantou Central Hospital, Shantou, Guangdong, China; ^5^ Product Development Department, Shantou Institute of Ultrasonic Instruments, Shantou, Guangdong, China; ^6^ The Breast Center, Cancer Hospital of Shantou University Medical College, Shantou, Guangdong, China

**Keywords:** computer-aided diagnosis, breast cancer, ultrasound, punch biopsy, needle segmentation

## Abstract

**Introduction:**

Ultrasound-guided needle biopsy is a commonly employed technique in modern medicine for obtaining tissue samples, such as those from breast tumors, for pathological analysis. However, it is limited by the low signal-to-noise ratio and the complex background of breast ultrasound imaging. In order to assist physicians in accurately performing needle biopsies on pathological tissues, minimize complications, and avoid damage to surrounding tissues, computer-aided needle segmentation and tracking has garnered increasing attention, with notable progress made in recent years. Nevertheless, challenges remain, including poor ultrasound image quality, high computational resource requirements, and various needle shape.

**Methods:**

This study introduces a novel Spatio-Temporal Memory Network designed for ultrasound-guided breast tumor biopsy. The proposed network integrates a hybrid encoder that employs CNN-Transformer architectures, along with an optical flow estimation method. From the Ultrasound Imaging Department at the First Affiliated Hospital of Shantou University, we developed a real-time segmentation dataset specifically designed for ultrasound-guided needle puncture procedures in breast tumors, which includes ultrasound biopsy video data collected from 11 patients.

**Results:**

Experimental results demonstrate that this model significantly outperforms existing methods in improving the positioning accuracy of needle and enhancing the tracking stability. Specifically, the performance metrics of the proposed model is as follows: IoU is 0.731, Dice is 0.817, Precision is 0.863, Recall is 0.803, and F1 score is 0.832. By advancing the precision of needle localization, this model contributes to enhanced reliability in ultrasound-guided breast tumor biopsy, ultimately supporting safer and more effective clinical outcomes.

**Discussion:**

The model proposed in this paper demonstrates robust performance in the computer-aided tracking and segmentation of biopsy needles in ultrasound imaging, specifically for ultrasound-guided breast tumor biopsy, offering dependable technical support for clinical procedures.

## 1 Introduction

In the realm of modern medicine, ultrasound-guided breast tumor biopsy is a cost-effective, convenient, and safe diagnostic method commonly employed for obtaining tissue samples for histological examination and pathological analysis (1). This technique aids physicians in confirming the origin and nature of breast lesions, as well as in monitoring disease progression (2). Ultrasound imaging is frequently employed to guide needle biopsy in real-time, enabling physicians to accurately navigate the needle to the target site while minimizing complications and preventing damage to surrounding tissues (2–5). However, the unique characteristics of ultrasound imaging, such as its low signal-to-noise ratio and dependence on the beam-to-needle orientation, introduce challenges in maintaining consistent visibility of the needle during procedures.

During the imaging process, those electronically controlled ultrasound beams which perpendicular to the needle produce strong specular reflections, thus enhancing needle visibility (6, 7). However, this optimal condition is often not maintained during the biopsy. When the needle direction is not perfectly perpendicular to the ultrasound beams or deviates from the ultrasound plane, it may result in unclear or completely invisible needle imaging (8). Furthermore, the needle’s visibility diminishes with increasing insertion depth due to the attenuation of ultrasound beams (9). These challenges are exacerbated by the needle’s small size, low contrast with surrounding tissues, and motion artifacts, which can mislead inexperienced physicians and increase the likelihood of inaccurate biopsy sampling and additional surgical risks (10).

To address these issues, during ultrasound-guided biopsy procedures where physicians manually manipulate the needle, real-time and precise computer-aided needle segmentation and tracking are essential. Such technologies can assist physicians in accurately locating the needle while adjusting the needle’s position and angle in real-time, thereby significantly improving both the safety and accuracy of the procedure. Particularly for clinical novices and early-career physicians, AI-assisted tools play a pivotal role in reducing technical barriers, increasing confidence, and enhancing procedural outcomes. By enabling real-time feedback, these systems ensure optimal needle trajectory and alignment, even under suboptimal imaging conditions.

Previous research has explored various approaches to needle segmentation and tracking in ultrasound images. Device-based solutions, such as electromagnetic trackers (11), have been proposed, but image-based methods are generally more suitable for clinical settings due to their ease of integration. Traditional computer vision and machine learning techniques based on image have been extensively investigated. For example, Novotny et al. (12) proposed using Principal Component Analysis (PCA) to integrate prior knowledge with ultrasound data for enhanced representation. Ding et al. (13) developed a template-based method for preprocessing ultrasound images to increase contrast between the needle tip and surrounding tissues, and applied the Gaussian Flow Lines (GFL) algorithm to detect the needle edge. Zhou and Qiu et al. (14, 15) proposed a 3D Hough transform method using distance metrics to optimize fitting results, while Kaya and Senel et al. (16, 17) introduced a two-stage Gabor filter method for needle tip localization and optimized estimation of insertion angles. Although these methods offer valuable insights, they are limited by their dependency on image quality, high computational demands, and sensitivity to variations in needle shape and size.

With rapid advancements in deep learning technology, its application in computer vision and medical image analysis has become increasingly widespread, leading to the emergence of deep learning methods for needle segmentation in ultrasound images. Geraldes and Rocha et al. (18, 19) used Multi-Layer Perceptrons (MLPs) to segment needle in 2D ultrasound images, demonstrating the feasibility of deep learning for detecting needle in challenging ultrasound images. Pourtaherian et al. (20) proposed an Orthogonal Plane Convolutional Neural Network (OPCNN) to detect needle positions in 3D ultrasound images. Yang et al. (21) introduced the VOI-CNNs model, which utilizes a three-plane convolutional neural network for needle segmentation. Lee et al. (22) developed a segmentation-based tracking model that integrates spatial and channel “Squeeze and Excitation” (scSE) modules, and Yang et al. (23) proposed a Direction-Fused Fully Convolutional Network (DF-FCN) to train models both along and perpendicular to the needle axis. While these methods reduce the need for manual intervention required by traditional approaches, they typically rely on large amounts of annotated training data and computational resources. To improve detection efficiency, Mwikirize et al. (24) proposed a region-based Fast R-CNN for detecting needle in 2D ultrasound images. Ronneberger et al. (25) introduced U-Net, a fully convolutional network that leverages multi-scale semantic information from ultrasound images to enhance detection accuracy. Additionally, many similar works have focused on detailed optimizations: Zhang et al. (26) proposed an attention-based U-Net for multi-needle segmentation and localization; Yang et al. (27) introduced a 3D patch-wise method to segment needle in 3D ultrasound images by dividing the 3D image into small blocks; and Bi et al. (28) developed a method to explicitly separate anatomical and domain features by calculating mutual information in the latent space, improving the generalization ability of segmentation models.

However, despite these advancements, existing deep learning methods for needle localization in ultrasound-guided procedures struggle to effectively extract features from ultrasound images with low signal-to-noise ratios and complex backgrounds. Moreover, they fail to leverage the inter-frame relationships present in needle video sequences, overlooking subtle displacements and morphological changes of the needle in complex tissue backgrounds. Consequently, these limitations result in insufficient localization accuracy and affect the safety and precision of clinical operations.

To address these limitations, we propose a novel Spatio-Temporal Needle Attention Network (STNAN), which achieves accurate segmentation, tracking, and prediction of the needle’s dynamic trajectory. Unlike conventional methods, STNAN leverages the inter-frame relational information inherent in ultrasound video sequences to enhance its tracking capabilities. Subsequently, we propose a feature encoder that integrates CNN and Transformer architectures, enabling the model to extract multi-scale features and local information from ultrasound images while capturing long-range dependencies across image sequences. Additionally, we design an innovative feature processing method that incorporates optical flow estimation to extract subtle displacement and morphological features of the needle in complex tissue environments. Furthermore, the proposed approach offers substantial potential for clinical implementation, particularly in assisting novice physicians, by providing additional support and enhancing operator confidence.

The main contributions of this paper are as follows:

1. To leverage the correlation information between frames in video sequences, we introduce a memory bank structure to store features extracted from ultrasound needle video sequences, thereby effectively processing and utilizing temporal information.
2. To address the challenges posed by ultrasound images with low signal-to-noise ratios and complex backgrounds, we propose a semantic feature encoder that integrates CNN and Transformer structures. This encoder not only extracts multi-scale features and local information from the images but also captures long-range dependencies within them.
3. To accurately capture and track the needle’s motion trajectory, we propose a feature processing method that integrates optical flow estimation, capable of extracting subtle displacements and morphological changes of the needle.

## 2 Materials and methods

**Figure 1:**
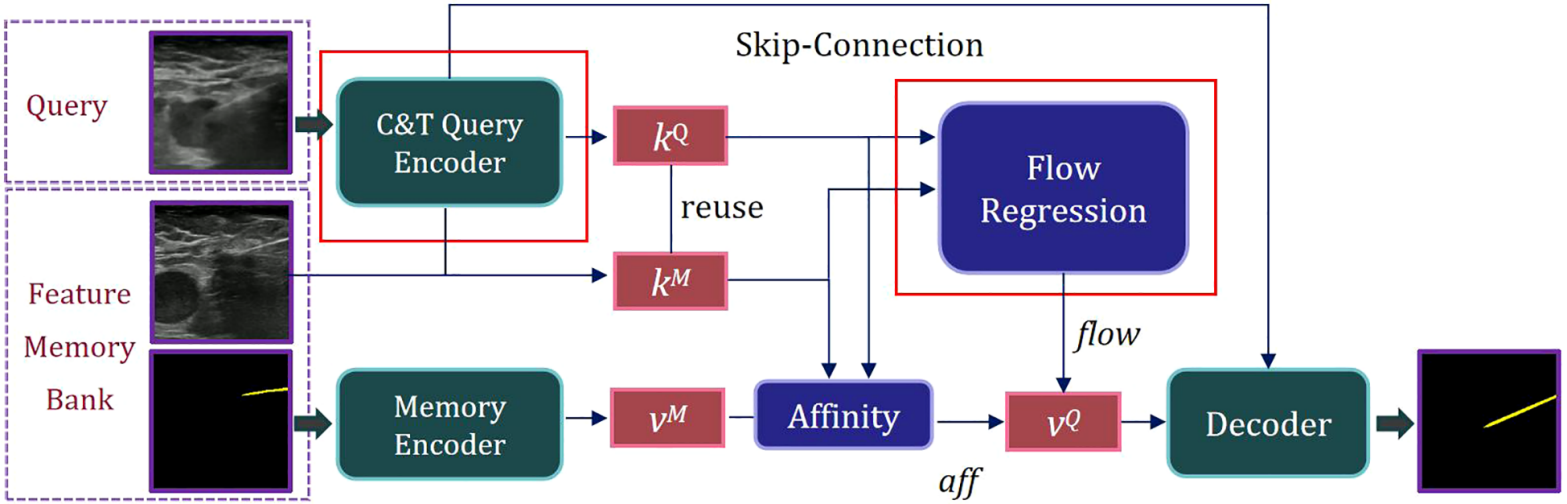
Overview of the proposed STNAN for needle segmentation.

The natural scene model Space-Time Correspondence Networks (STCN) provides an effective framework for capturing spatio-temporal correspondences (29). However, it is confronted with two challenges: due to substantial domain differences between medical and natural scene images, the basic feature extraction approach may be insufficient for capturing critical information in medical imaging, resulting in mismatched features. Additionally, common global matching patterns can lead the model to mistakenly segment distant objects with similar appearances. To address these challenges, we proposed Spatio-Temporal Needle Attention Network (STNAN) based on STCN, which employs the negative squared Euclidean distance to effectively model spatio-temporal correspondence, avoiding the need to re-encode mask features for each frame. The model utilizes a memory bank to store feature information from the previous frames. Furthermore, we introduce a novel hybrid architecture feature encoder and integrate optical flow information from adjacent frames as supplementary features to perceive motion information. The network structure is depicted in **Figure 1**.

STNAN takes a video sequence and the annotation of the first frame as input, processes the video frame by frame, and establishes a Feature Memory Bank. The network includes two encoders: C&T Query Encoder and Memory Encoder. The C&T Query Encoder takes the current frame of the ultrasound image as the query frame input, extracts the key feature of the query frame, denoted as *k^Q^*. Meanwhile the Memory Encoder takes the previous images and corresponding masks as input, extracts the associated value feature, denoted as *v^M^*.

During the sequence query process, features are extracted only once per frame. After completing a query, the key feature *k^Q^* of the current query frame is used as the key feature *k^M^* of the memory frame for the next query, reducing computational overhead. The key feature *k^Q^* of the query frame is compared with the key feature *k^M^* of the memory frame, and the affinity value *aff* between them is calculated using the negative squared Euclidean norm. The corresponding value feature *v^M^* is retrieved from the Feature Memory Bank. The key features *k^Q^* and corresponding *k^M^* are processed through our designed Optical Flow Regression module to obtain the optical flow vector *flow*. This vector, along with the affinity value *aff*, is utilized to calculate the value feature of the query frame, denoted as *v^Q^*. Finally, the mask of the current frame’s target object can be generated through the decoder.

### 2.1 Feature extraction with hybrid architecture of C&T query encoder

For ultrasound needle images characterized by low signal-to-noise ratios and complex backgrounds, fully extracting the needle’s features is essential for improving network accuracy. Convolutional Neural Networks (CNNs) are highly effective at extracting deep, discriminative features from image data, particularly excelling at capturing local information and multi-scale features. However, CNNs have limitations in handling long-range dependencies, and experience a significantly increase in computational complexity as network depth grows. On the other hand, Transformers effectively capture long-range dependencies in images through self-attention mechanisms but perform less sensitively in processing local structural details. Additionally, Transformers entail significant computational resources when dealing with high-resolution images and exhibit relatively weaker capabilities in extracting local feature.

**Figure 2:**
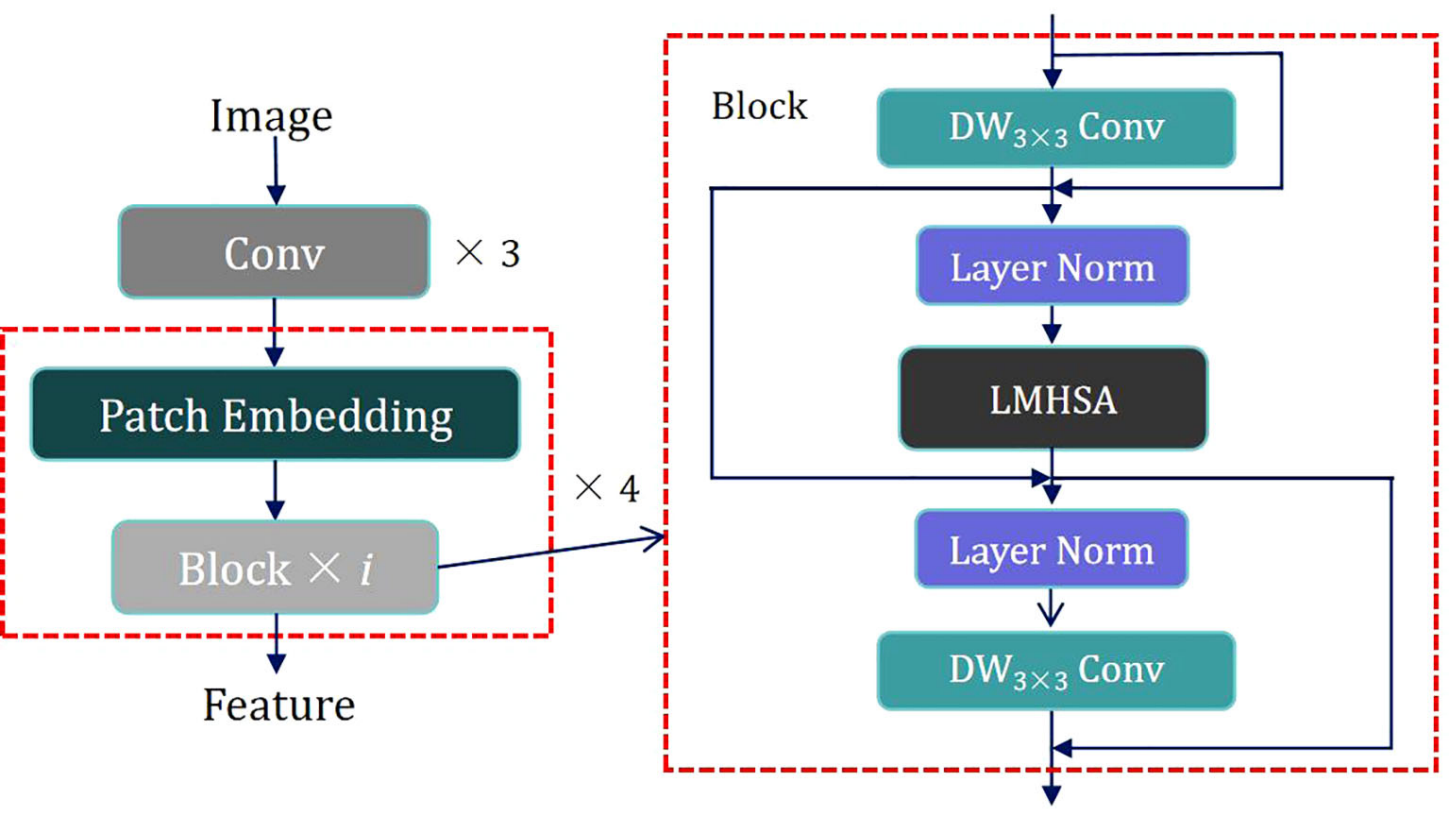
Scheme of the proposed Hybrid Architecture of C&T Query Encoder.

To capitalize on the strengths of both CNNs and Transformers, we propose a hybrid architecture called C&T Query Encoder, which integrates CNN and Transformer components. This architecture utilizes CNNs’ capabilities in local feature extraction and multi-scale information processing, alongside Transformers’ ability to capture long-range dependencies, thereby achieving more comprehensive and efficient feature extraction in image analysis tasks. Its structure is illustrated in **Figure 2**.

The design of the C&T Query Encoder follows these steps: The input image is first processed through three convolutional layers for initial processing, downsampling, and local feature extraction while preserving spatial information. The output of these initial convolutional layers is then fed into four combined modules, each consisting of a Patch Embedding layer and i Blocks. These modules are alternated to apply Self-Attention at various scales, capturing connections between different regions in the image. The Patch Embedding layer performs two-fold downsampling through a convolutional layer to extract low-resolution and multi-scale features. Each Block contains residual structures with both Depth-Wise Separable Convolution (DW Conv) and a Lightweight Multi-Head Self Attention module (LMHSA). These modules are interspersed with Layer Norm (LN), ensuring stable feature extraction.

DW Conv significantly reduces computational complexity and the number of parameters by separating the convolution operation into depthwise and pointwise convolutions, thereby enhancing computational efficiency. In each Block, we employ residual structures, initially implement a DW Conv with a 3 × 3 kernel size, followed by the integration of the LMHSA module, and subsequently reapply another DW Conv. This configuration effectively extracts local features from the feature map by combining the advantages of depthwise and pointwise convolutions. Additionally, the introduction of residual connections facilitates efficient gradient propagation throughout the network, thus enhancing training stability. The overall goal is to improve translation invariance in computer vision tasks while maintaining computational efficiency.

**Figure 3:**
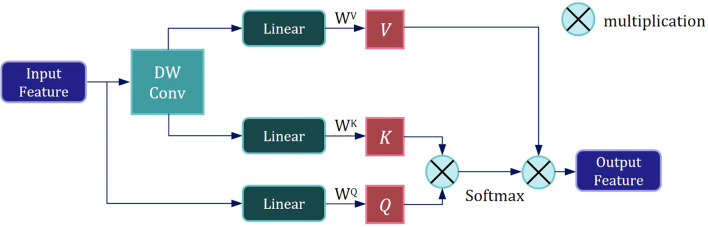
Scheme of the proposed Lightweight Multi-Head Self Attention module.

Following the first DW Conv module, Layer Normalization is applied to the feature map, enhancing training stability and convergence speed. The normalized feature map is then fed into the LMHSA module, as illustrated in **Figure 3**.

We employ a size-controllable DW Conv to reduce the resolution of the input feature, thereby decreasing the size of the *K* and *V* subspace feature maps and lowering the computational load of self-attention mechanism. The input feature is then mapped to three subspaces—*V*, *K*, and *Q*— using three distinct linear spatial mapping matrices *W^V^*, *W^K^*, and *W^Q^*, and the attention value is calculated as follows:


$$attention(Q,K,V)=Softmax(\frac{QK^{T}}{\sqrt{d}})V$$

where *d* represents the dimension of the key vector

After the LMHSA module, we further apply Layer Normalization to the feature map. This is followed by another DW Conv to process the featuremap, extracting local information.

Through the above design, the model can effectively extract semantic features, thus providing robust feature support for subsequent segmentation tasks.

### 2.2 Flow regression estimation based on search window

Deep stereo matching methods can leverage multi-view images to compute depth information, thereby improving the model’s ability to perceive the needle’s spatial position. Since stereo matching can be considered a specific case of optical flow, the matching cost learning in optical flow estimation is equally applicable to needle motion estimation. Optical flow estimation captures subtle motion information between adjacent frames, which is essential for detecting the needle’s minute displacements. By estimating optical flow, the model gains a deeper understanding of the needle’s motion patterns, enabling more precise trajectory tracking during segmentation.

In our deep stereo matching approach, we introduce a matching cost learning mechanism to enhance the accuracy of optical flow estimation by learning the matching cost between adjacent frames.

**Figure 4:**
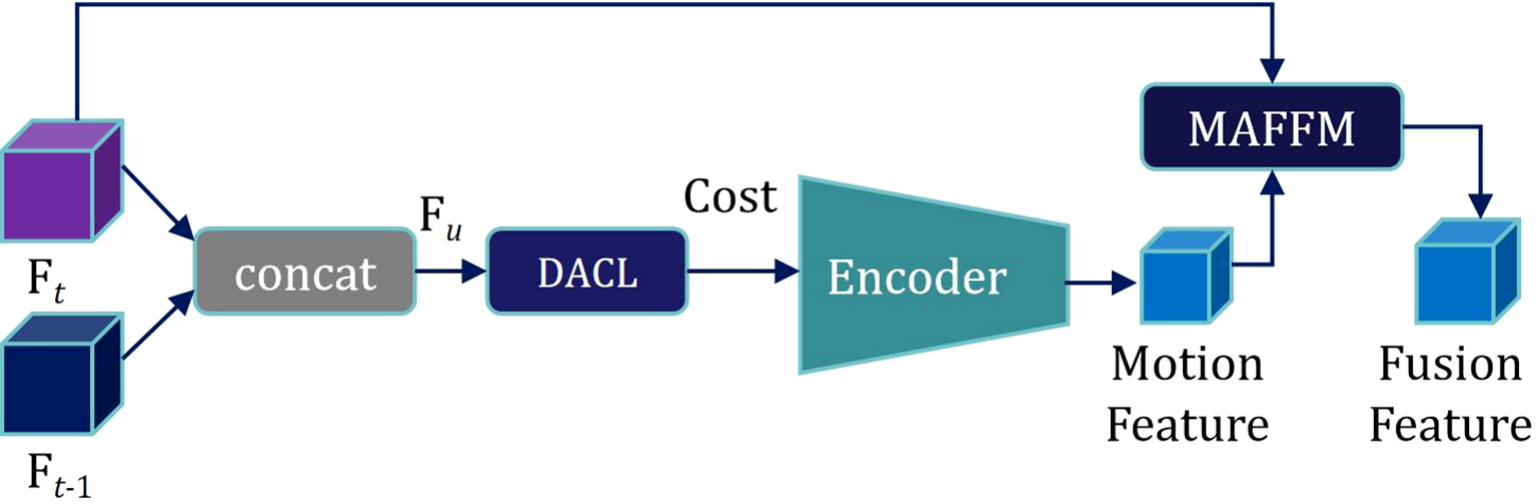
Scheme of the proposed feature processing method incorporating optical flow estimation.


As shown in **Figure 4**, the Semantic Feature *F_t_* of the current frame is superimposed with the features *F_t-1_* under each hypothesis to obtain U × V Semantic Fusion Feature Maps *F_u_(p)*:

$$F_{u}(p)=F_{t}(p)||F_{t-1}(p+u)$$

where *p* = (*x*, *y*) represents the original position coordinates, ‖ denotes superposition in the channel dimension, and u represents the displacement hypothesis.

**Figure 5:**
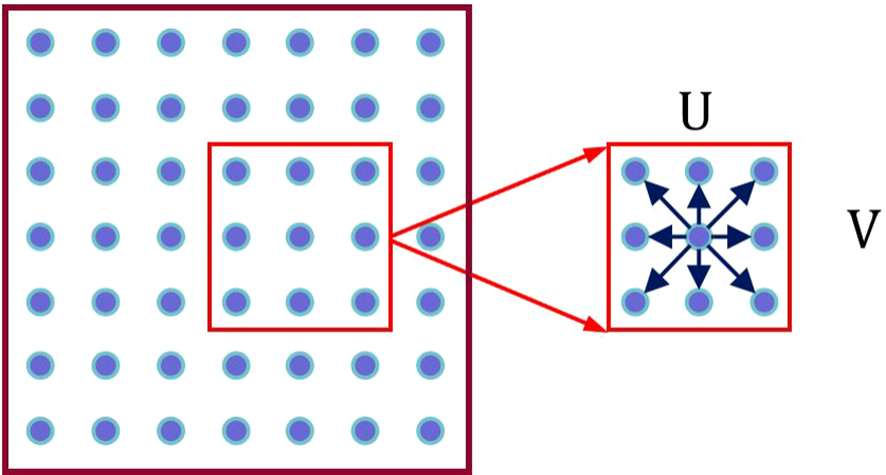
Illustration of the displacement search box.

In the feature map, for each pixel, we limit the scan matching range to a local window with determined scale containing U × V pixels. By hypothesizing various displacement directions of each pixel, we obtain corresponding *F_u_(p)*, as shown in **Figure 5**.

As seen in the **Figure 5**, each pixel has U × V displacement hypotheses.

On this basis, the semantic fusion feature map *F_u_(p)* is fed into the Displacement Aware Cost Learning (DACL) module. The DACL module comprises a series of convolutional and deconvolutional layers to extract features from *F_u_(p)*. Using a CNN-based 2D matching network, the module calculates local correlations and aggregates matching costs under U × V different displacement hypotheses, producing a comprehensive matching cost, denoted as *Cost*. Furthermore, the DACL module reweights the matching cost through a 1 × 1 convolution, calculated as follows:


$$Cost(u,p)=Conv_{1\times 1}(\sum_{u=(U,V)}M(F_{u}(p)))$$

where *M* represents the 2D matching network.

**Figure 6:**
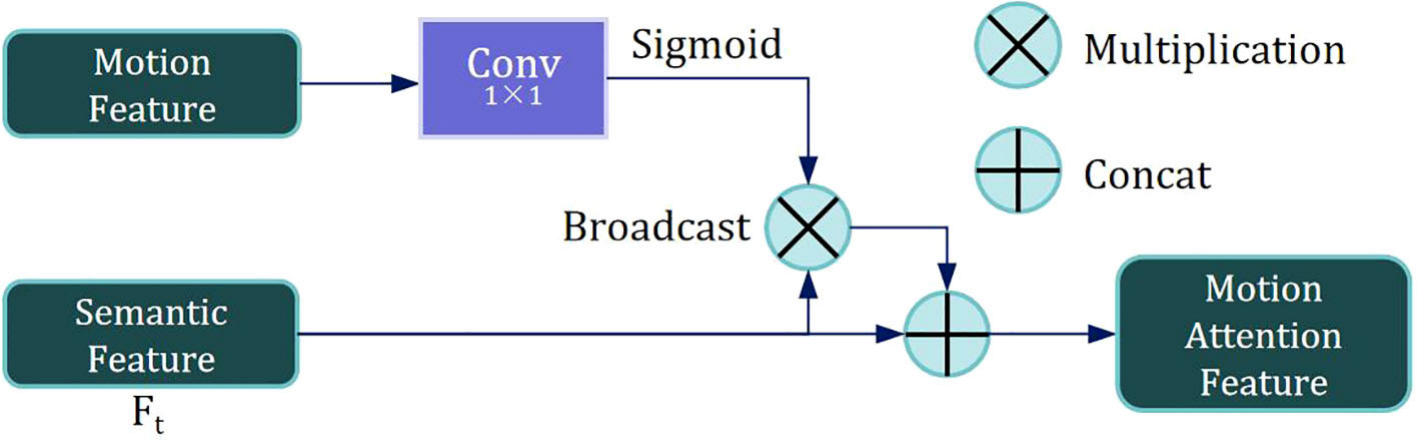
The structure of the Motion-attention based feature fusion module (MAFFM).

Subsequently, motion features from the previous and current frames are extracted by encoding. Motion features are then fused with the semantic feature *F_t_* of the current frame through the Motion-attention Based Feature Fusion Module (MAFFM), as illustrated in **Figure 6**.

We begin by applying a 1 × 1 convolution to reduce the dimensionality of the Motion Feature, followed by smoothing them using a Sigmoid activation function. A broadcast mechanism is then employed to reweight the Semantic Feature *F_t_* of the current frame. Finally, we superimpose *F_t_* onto itself to obtain a Motion Attention Feature, which is utilized for subsequent mask propagation. This design enables the model to effectively exploit inter-frame relationships within the needle sequence, allowing it to accurately capture the needle’s motion trajectory.

### 2.3 Real-time ultrasound needle dataset

In the field of ultrasound-guided biopsy, particularly for breast tumor puncture, there is a significant shortage of publicly available datasets for needle segmentation. As a result, most existing studies rely on non-public datasets derived from non-human samples. These datasets often do not accurately reflect real surgical conditions and typically focus on single-frame images, overlooking the continuity of needle movement during the biopsy process. To address this gap, in collaboration with physicians from the Ultrasound Imaging Department at the First Affiliated Hospital of Shantou University, we collected ultrasound biopsy video data from 11 patients of varying ages, creating a real-time segmentation dataset specifically tailored for ultrasound-guided needle puncture in breast tumors. Each video was recorded at a frame rate of 25 frames per second. The institutional review board approved this study, and the requirement to obtain informed consent was waived (approval number: B-2022-182).

To ensure temporal continuity and reduce redundant features, we extracted frames at random intervals of 5 to 10 frames, selecting approximately 250 consecutive frames from each video. Each frame contains a single needle, annotated by experienced ultrasound imaging physicians to obtain mask images as dataset labels. We used nine video frame sequences (approximately 2,250 images) for the training set and the remaining two sequences (approximately 500 images) for the test set. All images were uniformly resized to 448 × 448 pixels.

### 2.4 Loss function and evaluation metrics

Biopsy needle in ultrasound images appear as thin, elongated straight lines and are significantly outnumbered by background pixels. To address this issue of imbalanced samples, we adopt Binary Cross Entropy (BCE) as the loss function. The calculation of BCE is as follows:


$$L_{BCE}=-(y*\log(p)+(1-y)*\log(1-p))$$

where *y* represents the true label of the sample (1 for biopsy needle, 0 for background), and *p* represents the probability that the model predicts the sample as a biopsy needle.

To comprehensively assess the segmentation performance, we use several evaluation metrics, including Intersection over Union, Dice coefficient, Precision, Recall, and F1-score.

The calculation formulas for *IoU*, *Dice*, *Precision*, *Recall*, and *F1-score* are as follows:


$$IoU=\frac{TP}{TP+FP+FN}$$

$$Dice=\frac{2TP}{2TP+FP+FN}$$

$$Precision=\frac{TP}{TP+FP}$$

$$Recall=\frac{TP}{TP+FN}$$

$$F_{1}-score=\frac{2*Precision*Recall}{Precision+Recall}$$

Considering the real-time requirements of ultrasound-guided biopsy procedures, we measured the inference speed of the model on the test set, recording both the number of frames segmented per second and the average time required for segmenting each frame.

### 2.5 Training parameters and training method settings

Our model was trained on an NVIDIA GeForce RTX 3090 GPU using the Adaptive Moment Estimation (Adam) optimizer. The training was conducted with a batch size of 4, an initial learning rate of 10^-5^, and a learning rate decay factor of 0.2.

To enhance the continuity of the model’s segmentation and tracking performance, we employed the following training strategy: During each iteration, four video sequences were randomly selected from the training set and we sequentially select five images as training samples, with the first image providing the ground truth mask label. Memory features were extracted from the first frame and its corresponding mask label to predict the mask of the second frame, and the features from both frames were stored in the memory repository. These fused memory features were then used to predict the masks for subsequent frames in sequence. For all frames except the first, the predicted masks were compared with the ground truth labels for loss calculation, followed by backpropagation and parameter updates in the network. To improve the model’s robustness, we randomly reverse the temporal order of the five continuous frames during training, enabling the model to learn both forward and backward predictions.

To prevent redundant memory features and maintain efficient inference speed, we adopted the default memory storage interval of STCN, storing semantic and mask features every 5 frames.

## 3 Results

### 3.1 Parameter optimization experiments

We introduces the C&T Query Encoder, which allows for the stacking of a variable number of Blocks, thereby facilitating a balance between model accuracy and inference speed. We systematically explored the impact of the number of Blocks *i* in the C&T Query Encoder on model performance and validated the performance of the STCN+C&T model under various configurations through experimentation. **Table 1** presents the performance metrics of the STCN+C&T model when varying numbers of Blocks are stacked in the C&T Query Encoder. In terms of the performance metrics, multiple standard metrics including IoU, Dice, Precision, Recall, F1 scores and FPS are used for evaluating image segmentation tasks.

**Table 1:** Performance comparison of STCN+C&T model with different numbers of blocks.

The number of blocks	IoU	Dice	Precision	Recall	F1	FPS
(2, 2, 6, 2)	0.718	0.806	0.840	**0.803**	**0.821**	**60.17**
(2, 2, 10, 2)	0.712	0.803	0.838	0.799	0.818	55.41
(3, 3, 12, 3)	0.709	0.802	0.845	0.790	0.816	52.53
(3, 3, 16, 3)	**0.720**	**0.809**	**0.854**	0.790	0.820	46.52

The bold values provided in the table below indicate the best-performing metrics in comparison.

In **Table 1**, the first column represents the number of Blocks in the four stacked structures of the C&T Query Encoder. As the number of Blocks increases, the IoU and Dice scores of the STNAN model improve, while inference speed decreases correspondingly. The experiments reveal that when the number of Blocks is set to 3, 3, 16, and 3 respectively, the STCN+C&T model achieves optimal performance on the test set, with an IoU of 0.720, Dice of 0.809, F1 score of 0.820, and FPS of 46.52, significantly surpassing the 25 FPS ultrasound biopsy video data provided by the hospital, thereby meeting real-time operational requirements.

Furthermore, we investigated the effect of varying displacement search window sizes in optical flow estimation on model performance. On a semantic feature map with a size of 12 × 12, we set various displacement search window sizes and conducted experiments under the optimal Block configuration, with the results shown in **Table 2**.

**Table 2:** Performance of STNAN with different displacement search window sizes.

Size of window (U, V)	IoU	Dice	Precision	Recall	F1	FPS
U=5, V=5	0.712	0.801	0.855	0.775	0.813	**38.59**
U=7, V=7	0.722	0.810	0.851	0.798	0.824	37.74
**U=9, V=9**	**0.731**	**0.817**	**0.863**	**0.803**	**0.832**	35.57
U=11, V=11	0.720	0.809	0.852	0.796	0.823	30.26

The bold values provided in the table below indicate the best-performing metrics in comparison.

Since the displacement search window is centered on each pixel and has an odd-numbered side length, we implemented four window size configurations: 5 × 5, 7 × 7, 9 × 9 and 11 × 11. The results indicate that the performance of the model improves when the displacement search window size increases from 5 × 5 to 9 × 9; When the size continues to increase to 9 × 9, the model effect decreases. We speculate that this decline occurs because the needle displacement between some frames is large, and a smaller search window cannot adequately capture these variations, resulting in some needle pixels in the past frames not matching the correct points in the current frame. As the search window size continues to increase, although more information can be captured, it may also lead to matching noise points or irrelevant points in the background, thereby reducing the accuracy of the matching. Ultimately, when the displacement search window size is 9 × 9, the model achieves its optimal performance, which can also satisfy real-time operational requirements.

### 3.2 Ablation study

This paper introduces several improvements to the original STCN model, proposing the STNAN model. The effectiveness of each improvement module to improve the model’s performance was validated through a series of experiments. Specifically, we compared the performance of the original STCN model with that of the model incorporating each improvement module, with the results shown in **Table 3**.

**Table 3:** Ablation study results of the STNAN model.

Model	IoU	Dice	Precision	Recall	F1	FPS
STCN	0.696	0.793	0.861	0.761	0.808	**60.79**
STCN+C&T	0.720	0.809	0.854	0.790	0.820	46.52
**STCN+C&T+FR(STNAN)**	**0.731**	**0.817**	**0.863**	**0.803**	**0.832**	35.57

The bold values provided in the table below indicate the best-performing metrics in comparison.

The performance metrics of the original STCN model is as follows: IoU is 0.696, Dice is 0.793, Precision is 0.861, Recall is 0.761, F1 score is 0.808, and FPS is 60.79, with a single-frame segmentation time of 0.016 seconds.

After adding C&T Encoder and FR modules, significant improvements are demonstrated across recall (0.761 vs 0.803), precision (0.861 vs 0.863), IOU (0.696 vs 0.731) and Dice(0.793 vs 0.863).In conclusion, the proposed improvement strategies in this paper have significantly enhanced the performance of the model in ultrasound needle tracking and segmentation tasks.

### 3.3 Comparative experiments

**Table 4** presents the results of the proposed STNAN against all the compared methods over the datasets. Comparisons between our STNAN and recently favored frameworks for medical image segmentation were conducted, benchmarking against convolutional baseline models such as U-Net, VGG16 and FCN32. We further perform comparison against the STCN segmentation networks.

**Table 4:** Comparison with various models with respect to efficiency and segmentation metrics.

Model	IOU	Dice	Precision	Recall	F1	FPS
PSPNet	0.233	0.348	0.579	0.291	0.387	29.15
FCN32	0.563	0.689	0.762	0.676	0.716	19.93
UNet	0.511	0.623	0.716	0.660	0.687	23.63
SegNet	0.399	0.548	0.621	0.527	0.570	15.50
VGG16+UNet	0.508	0.649	0.789	0.667	0.723	22.90
ResNet50+FCN32	0.566	0.697	0.737	0.684	0.710	9.96
STCN	0.696	0.793	0.861	0.761	0.808	**60.79**
**STNAN(Ours)**	**0.731**	**0.817**	**0.863**	**0.803**	**0.832**	35.57

The bold values provided in the table below indicate the best-performing metrics in comparison.

The results demonstrate that our STNAN model surpasses the performance of all other methodologies. This highlights the significant advantages of STNAN in terms of segmentation accuracy and overall quality, indicating its strong suitability for meeting the clinical requirements of biopsy procedures.

**Figure 7:**
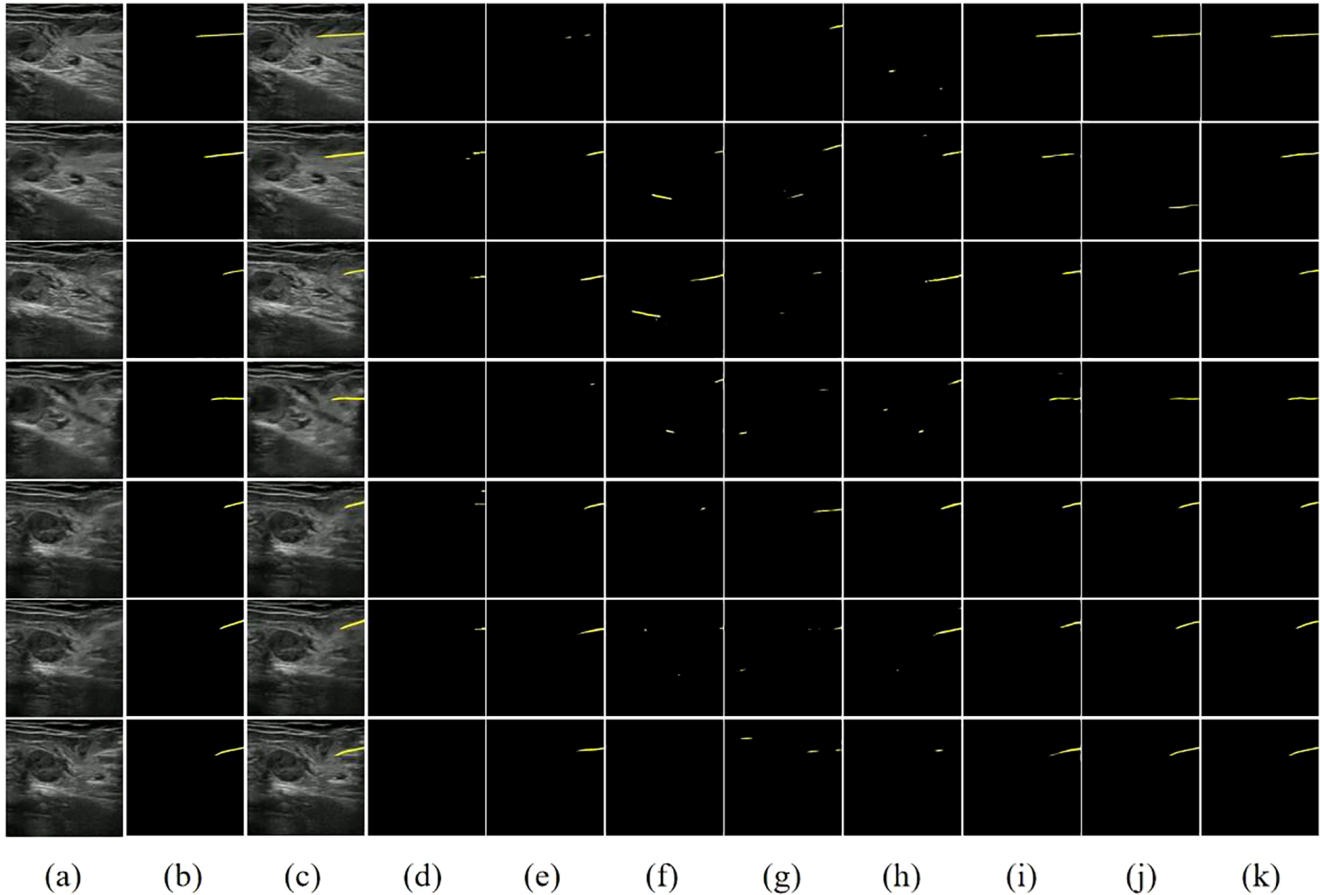
Visual comparison of different methods on our dataset. **(A)** Image. **(B)** Ground Truth. **(C)** Image+GT. **(D)** PSPNet. **(E)** FCN32. **(F)** UNet. **(G)** SegNet. **(H)** VGG16+UNet. **(I)** ResNet50+FCN32. **(J)** STCN. **(K)** STNAN. Yellow indicates the prediction of needle.

To visually illustrate the performance differences between various models in handling ultrasound needle tracking and segmentation tasks, we selected several ultrasound needle images from the test set in sequential order, emphasizing cases where the grayscale visibility of the biopsy needle is relatively weak. Furthermore, we conducted a visual analysis of each model’s segmentation and tracking results, as depicted in **Figure 7**.

The first two columns of **Figure 7** display the original ultrasound images from the test set alongside their corresponding ground truth labels. The third column displays the superimposed ultrasound images along with ground truth data, while the remaining columns show the segmentation results of each model. Classic semantic segmentation models often struggle with challenging ultrasound needle tracking and segmentation tasks, frequently failing to accurately segment the needle and tenting to misidentify needle-like tissues in the image as the needle, resulting in an increased incidence of false positives in the segmentation results. The STCN model considers the spatio-temporal correlation between frames, improving the segmentation results to some extent. However, it still suffers from false positives due to its reliance on global matching techniques, which may undermine its effectiveness during clinical procedures.

In contrast, our STNAN model demonstrates superior performance in accurately identifying and tracking needle while maintaining a lower rate of false positives. It is attributed to the proposed semantic feature encoder for low signal-to-noise ratio ultrasound images and the optical flow regression estimation method. These advancements make STNAN highly promising for real-world clinical applications, as it effectively minimizes false positives while achieving precise segmentation of the puncture needle.

## 4 Discussion

This paper introduces a novel Spatio-Temporal Needle Attention Network (STNAN) to address key challenges in ultrasound-guided needle biopsy. By integrating Convolutional Neural Networks (CNNs) and Transformer architectures, STNAN effectively extracts multi-scale features and captures long-range dependencies. Additionally, the model incorporates an optical flow estimation mechanism to detect subtle displacements and morphological changes of the needle in complex tissue environments. Experimental results demonstrate that STNAN significantly outperforms existing methods in enhancing the accuracy and continuity of needle localization, thereby improving both the safety and precision of clinical procedures.

The proposed model is designed as an AI-assisted tool to support less experienced physicians during ultrasound-guided biopsy procedures. In practice, needle visibility is often compromised due to factors such as suboptimal angles, deviations from the ultrasound plane, and signal attenuation at greater depths, which pose significant challenges for novice operators. STNAN provides real-time needle tracking and segmentation, offering additional guidance and improving operator confidence.

The dataset used to train STNAN consisted of 11 patients, yielding approximately 2750 image frames. During model training, STNAN achieved convergence without signs of overfitting, demonstrating that the data was sufficient for learning key features of needle localization. However, we acknowledge that expanding the dataset could further enhance the model’s generalizability, especially when applied to more diverse clinical scenarios.

STNAN shows excellent performance in ultrasound-guided needle localization tasks, providing robust technical support for clinical operations. In future work, we will focus on further optimizing the model’s real-time performance and exploring its broader applications in ultrasound-guided interventions. Expanding training datasets with diverse clinical cases is expected to improve the model’s generalizability, and advanced techniques such as data augmentation and transfer learning will be employed to optimize model performance. Additionally, incorporating trajectory prediction features could further assist operators in planning needle movements, paving the way for safer and more precise procedures in clinical practice.

## Data Availability

The dataset fully protects patient privacy, has been approved by the ethics committee, and is intended for academic research only. Requests to access the datasets should be directed to Zhemin Zhuang, zmzhuang@stu.edu.cn.
